# Registered Nurses' experiences of reading and using research for work and education: a qualitative research study

**DOI:** 10.1186/s12912-022-00877-3

**Published:** 2022-05-12

**Authors:** Sonia Hines, Joanne Ramsbotham, Fiona Coyer

**Affiliations:** 1 Flinders SA & NT, College of Medicine and Public Health, Centre for Remote Health, Flinders University, 5 Skinner St, The Gap, Alice Springs, NT 0870 Australia; 2 School of Nursing, Queensland University of Technology, Victoria Park Rd, Kelvin Grove, Brisbane, Qld Australia; 3 Royal Brisbane and Women’s Hospital, Butterfield St, Herston, Qld 4029 Australia

**Keywords:** Nursing, Research, Research literacy, Focus groups, Qualitative research, Mathematics anxiety

## Abstract

**Background:**

Considerable resources have been expended, both in universities and health workplaces to improve nurses' abilities to interact with research and research literature to enable their engagement with evidence-based practice. Despite these efforts, a considerable number of nurses experience difficulty with research literature and are reluctant to use it in practice.

**Aims:**

This study aimed to explore the experiences and perceptions of Registered Nurses when they have been required to read and understand research literature for work or education.

**Design:**

A qualitative descriptive study using online and in-person focus groups.

**Methods:**

Focus groups (online and in-person) were conducted between June and November 2020. Forty participants were included. We used focus group recordings and field notes to collect data. Transcribed records of these focus groups were coded on the basis of similarity of meaning and then subjected to thematic analysis.

**Results:**

Three distinct themes were identified from the data: 'coming into learning about research', fitting research into the reality of nursing life', and 'working towards using research.' Participants described their early experiences in learning about research, experiences both positive and negative in integrating research into practice, and their personal strategies for reading and using research, particularly in the context of significant anxiety about understanding the content of methods and results sections of quantitative research articles.

**Conclusion:**

This study goes beyond the barriers and facilitators dichotomy that has been the majority of the conversation about nurses' evidence-based practice engagement previously, and explores the issues underlying aversion to research literature. Many nurses struggle with the language, numbers, and/or statistics used in research and this requires educational interventions suited to the problem and the population.

## Introduction

Reading and using research is integral to evidence-based practice and therefore to nursing [1]. It is known, however, that many nurses avoid engagement with research literature and evidence-based practice (EBP) for a variety of reasons [2]. Positive attitudes to EBP, involvement in research education and activities, regular journal reading, and higher levels of education have been found to be associated with higher levels of EBP engagement [3]. These positive EBP attitudes may indicate that past experiences and associated feelings about research are a more important factor than perhaps the literature would indicate.

Considerable resources have been expended, both in universities and health workplaces to improve nurses' abilities to interact with research and research literature. Most university nursing degree courses around the world include research education in some form as part of their undergraduate curriculum, however in practice there continue to be nurses who are reluctant to engage with evidence-based practice and research utilization [4]. Globally, the World Health Organization (WHO) identifies nurse graduate attributes that includes the ability to demonstrate the use of evidence in practice [5]. This research, which is part of a larger body of work on nurses' research literacy, intends to describe RNs' perspectives on reading and using research in practice and education, to understand their feelings about this activity and to generate new knowledge about their behavior in this area of practice.

## Background

A qualitative systematic review of 11 studies has identified a broad range of emotional responses nurses may experience when interacting with research literature including negative feelings such as discomfort, irritation, frustration and vulnerability [6]. While this small body of qualitative research describes the barriers to nurses' research utilization in practice, a notable gap in understanding the experiences that have led to forming those emotional reactions has been identified.

The requirements for Registered Nurses are clear – they are expected to be able to participate in evidence-based practice and this requires them to be research literate – able to read and understand publications that use research language – but what is equally clear is that nurses find evidence-based practice difficult, challenging or even impossible [7–9].

In addition to factors such as organizational characteristics and pressures [10] it has been hypothesized that nurses experience difficulty understanding the language used in research literature [9]. Difficulties with language, however, may not be the only issue at the root of this problem. Nurses learn a great many specialist terms in their careers, quickly becoming familiar with the particular language used in different clinical, community and other specialist areas, so it seems unlikely that research language alone is the problem. It may be that there are particular feelings and experiences specifically attached to research literature that deter nurses from engaging with it, or other factors affecting their engagement. Much is known about barriers to EBP, but less is known about nurses' experiences and feelings about research in the context of their lives and careers.

## Methods

### Aims

The aim of this study was to explore the experiences and perceptions of registered nurses when they have been required to read and understand research literature as part of work or educational activities chiefly, to describe their feelings about this activity, to understand the relationship between these experiences and participants' willingness to engage in activities that require interaction with research literature and their experiences with those activities.

### Research question

This study was designed to answer the question, "How do registered nurses experience and perceive reading and using research for work and education?".

### Design

This study employed a qualitative descriptive design, as described by Sandelowski [11], Milne [12], Lambert [13], and Kim [14]. The aim of the qualitative descriptive design, according to Lambert, is to comprehensively summarize particular events experienced by individuals [13]. Used widely in nursing due to its pragmatic, simple approach, qualitative descriptive research seeks to understand experiences and perceptions without transforming them beyond recognition [15]. The qualitative descriptive design was derived from the interpretivist research paradigm which holds that reality and truth are socially constructed and that complex phenomena can have many interpretations [16].

Qualitative descriptive studies, considered a form of naturalistic inquiry [17], use straightforward methods of data collection, such as focus groups, to elicit information about participant experiences and so this methodology is most suitable for research questions such as those being posed in this study. This design is categorized by minimal transformation of the data, and to this end we attempted to utilize the participants' own voices as much as possible to convey their experiences as they described them [14].

In this study, we conducted a series of online and in-person focus groups utilizing semi-structured interviews to collect participants' responses to open-ended questions and prompts from the researcher about their experiences and perceptions. Focus groups, due to their inherently social nature, are ideal for revealing attitudes, beliefs and experiences.

### Sample/participants

The study protocol planned for a sample size of 75 registered nurses, however data saturation was reached at 40 participants and so recruitment was ceased. Sampling was not purposive, and any interested registered nurse was eligible to volunteer to participate.

The study population was planned to be drawn from registered nurses attending educational short courses or sessions at the study location (a center for education and research in a remote Australian town), however this was disrupted by the COVID-19 pandemic and travel and contact restrictions meant that in-person short courses and other education were moved to online delivery, preventing recruitment for in-person data collection, except for five participants for one focus group. Additional participants were then recruited to participate in online focus groups using nursing forum posts, social media, email, and personal contacts. Eligible participants were any adult person holding a current nursing registration with AHPRA (Australian Health Professional Regulation Agency), currently practicing in any health setting and with any educational background.

### Data collection

Data were collected between June and October of 2020. Online focus groups were conducted using Zoom video-conferencing software, which enabled video as well as audio capture of participants' interactions. Video-conferencing supported participant to participant interactions, as well as participant to researcher, and moderately replicated the strength of the social elements of an in-person focus group. The single in-person focus group was audio-recorded only, but field notes were recorded. Post-interview field notes were also recorded for the online focus groups. Fourteen focus groups were scheduled, with 45 min allocated to each. Following their completion of the consent form, participants were contacted with a range of focus group times to choose from and once three to six participants had chosen the same time slot, the group time was confirmed and took place. In three cases, scheduled participants did not attend or advise their inability to attend, and so the data collection proceeded with only one participant.

Expectations for the group in terms of turn-taking, disagreements and politeness were discussed at the start of each group's session. Focus groups each generally took 30–45 min to discuss the questions in the interview guide, although occasionally more time was taken due to lively conversation.

**Fig. 1:**
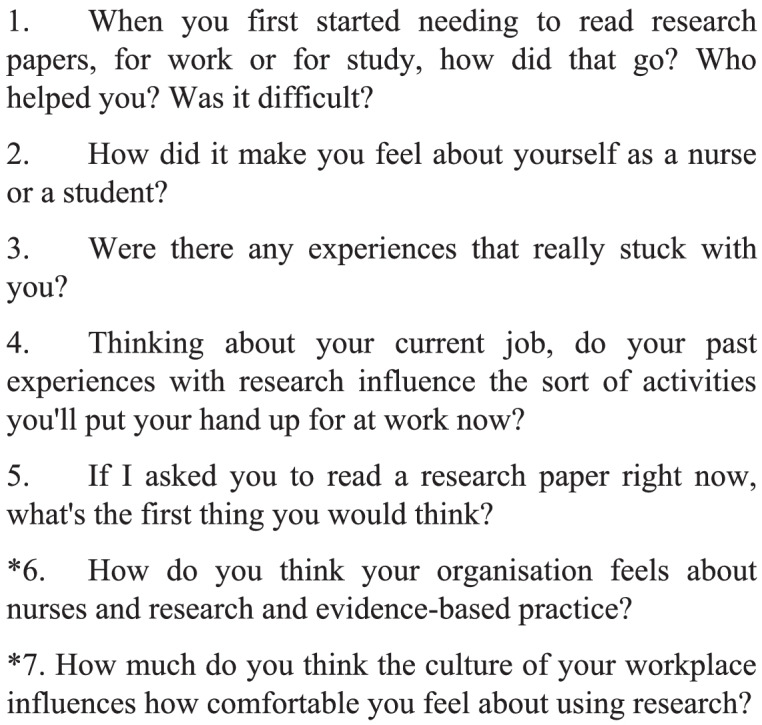
Interview guide. Items with asterisks* were added iteratively

The interview guide (Fig. 1) was developed by the researchers at the beginning of the study and changed iteratively over the course of the interviews in response to the discussions and two more questions were added. Questions in the interview guide were designed to answer the research question, and influenced by Melnyk's work on EBP in organizations, and EBP education [18–20] as well as the researchers' previous work in this field. The primary researcher was the only interviewer.

### Data analysis

This study used the six stage thematic analysis process recommended by Braun and Clark [21]:

1. After verbatim transcription of the audio recordings by a professional transcription service, the first author spent considerable time reading the transcripts and becoming familiar with the data.
2. Transcripts were entered into NVivo 12 (QSR International) which was used to aid thematic analysis. Initial codes were developed from both meaning and context by the PI at a semantic level of meaning. The codes were checked by the associate investigators to improve dependability.
3. Codes were then categorized into groups on the basis of patterns of similar meanings.
4. Categorization into themes and subthemes was achieved through repeated readings of the transcripts and considering the meaning of participants' statements. The associate investigators checked and gave input on the themes and subthemes at this stage.
5. The themes and subthemes were named in an iterative process that involved repeated readings and returns to the participant data to select the appropriate illustrative quotes which were then used verbatim to convey participants experiences and perceptions.
6. The sixth and final stage involved writing up the data, deciding on the order the themes and subthemes would be presented and making final decisions about how the story of the research might best be told. At this stage, participant names were replaced with pseudonyms to preserve confidentiality.

## Rigor

### Reflexivity

The primary researcher SH is a registered nurse coming from a professional background in nursing research and education, particularly focusing on evidence-based practice and research capability. Reflecting on her experiences teaching and learning about research and EBP, she needed to recognize her biases and prior assumptions regarding the root causes of disengagement with research and EBP literature, acknowledging these in discussions and making space for participants to relate their own experiences.

### Trustworthiness

The trustworthiness of this research was enhanced through careful attention to credibility, transferability, dependability, confirmability, auditability, and reflexivity [22, 23]. The credibility and auditability of this study was enhanced by the use of extensive record keeping for the field notes, recordings, transcripts, and coding. Decisions about coding and data management were clearly documented. After each focus group field notes were recorded and checked against the recording.

Transferability and authenticity [24] have been addressed by recording and reporting detailed 'thick' descriptions of the interactions and discussions in each focus group. While qualitative research is not precisely transferable, there are similarities between many kinds of human experiences and readers of the research may recognize the findings as transferable to their own context, particularly as we have included the participants' own words as much as practicable [22].

Dependability in qualitative research is similar to the concept of reliability in quantitative research [25]. In this study we have ensured the research has been accurately reported, that decisions were documented and so are able to be clearly auditable. The use of an interview guide to ensure the same questions are asked of each focus group was also designed to increase the dependability of the study. The confirmability of the study will be established when the above methods for achieving credibility, transferability and dependability have been enacted [22]. All data related to the study has been retained: focus group recordings, transcripts, field notes, coding decisions, the codebook, and NVivo files. Completeness of reporting was ensured by following the Consolidated Criteria for Reporting Qualitative Research (COREQ) guideline [26].

## Findings

### Participant descriptions

Initially 53 registered nurses (RN) volunteered and signed a consent to participate, however not all responded to the contact emails to arrange a focus group time or were able to find a suitable time to participate, and so 40 registered nurses completed the study in 14 focus groups of 1–6 participants. All focus groups were planned to be at least three participants in addition to the investigator, however last-minute cancellations meant that was not always possible and three focus groups proceeded with only one participant and the researcher conversing.

All participants were registered nurses licensed to practice in Australia, located in every state and territory of Australia in a variety of urban (*n* = 23), rural and remote areas (*n* = 17). Most participants were female, and their ages ranged from 24 – 65 years. Participants were working in a wide range of clinical settings including emergency department (*n* = 7), medical-surgical (*n* = 7), intensive and critical care (*n* = 5), mental health (*n* = 5), perioperative services (*n* = 3), oncology (*n* = 3), remote area nursing (*n* = 3), family nursing (*n* = 2), pediatrics (*n* = 1), occupational health (*n* = 1), community nursing (*n* = 1), rehabilitation (*n* = 1), and Aboriginal health (*n* = 1). All spoke fluent English, as required for nursing registration in Australia [27], however several spoke English as an additional language. Most were very experienced in their nursing career, with an average length of nursing experience of over 20 years and the majority of participants had a postgraduate level of education (Table 1).

**Table 1:** Participant Demographics

**Total**	*N* = 40					
Age	Mean 47.5 (SD: 10.27)					
Gender	Female *n* = 35			Male *n* = 5		
Length of Nursing Experience	Mean 21.27 (SD: 12.3)					
Highest Qualification	Hospital certificate /diploma	Bachelor degree	Graduate certificate/ diploma		Masters (including research)	PhD
N	2	13	10		13	2

### Themes

The 14 focus groups with 40 participants yielded three themes: 'Coming into learning about research', 'Fitting research into the reality of nursing life', and 'Working towards using research' and ten subthemes (Table 2). All participant names used here have been pseudonymized. Pseudonyms, ages and length of RN career are provided in parentheses with each participant quote to give further context to participants' responses.

**Table 2:** Themes

Theme	Subthemes	Elements
Coming into learning about research	Early experiences	Feeling lost
	Help with learning	Not knowing how or if to critique
		Courses, subjects, resources, programs, access to articles, teaching and learning strategies, educators, peers
Fitting research into the reality of nursing life	Organisational issues	Work culture, resources, staffing, roadblocks, disinterest, supportiveness, valuing research
	Interpersonal issues	Hostility, gate-keeping, apathy and reluctance, support, assistance, feeling out of place
	Confidence	Feeling capable and competent dealing with research, feelings about interacting with research
Working towards using research	Approaches to reading and understanding research	Selective reading of research papers, reading strategies
	Using research	Improving practice with research
	Mathematics difficulties	Numerical results, statistics, symbols, as a focus for problems understanding research
	Research language	Research-specific terms as a focus for problems understanding research

### Theme 1: Coming into learning about research

Given our interest in nurses' early learning experiences regarding research, a significant part of each group discussion focused on participants' first encounters with research. Participants had come to nursing from a variety of paths; as school-leavers, mature-age students changing careers or entering the workforce at a later age, so they had a range of educational and life-skills preparation as they entered nursing. Some participants had begun nursing prior to tertiary nursing education implementation, having been trained in hospitals, and this also impacted on their experiences of learning about research even if they had completed tertiary studies at a later time.

#### Early experiences

Most of our participants had begun their careers when nurse education was very different from today, some in the early days of university education and some through the hospital training system. This time gap had an impact on the recall of these early events for some participants but for others the memories of their experiences were very clear. Participants described, some with laughter, their initial feelings when first faced with learning about research, either in their undergraduate nursing degree or subsequent graduate level studies, conveying a range of reactions:

When I was first exposed to research as an undergrad, I was horrified (Jack, 55, RN 26 years), I mean it was really good. I loved it, but it was a very steep learning curve (Anna, 59, RN 13 years), and, I didn't really take any interest in articles until I started my first Masters (Joy, 52, RN 33 years).

Difficulty understanding the concepts and feeling lost were common experiences for these nurses as they began to learn about research. Using self-deprecating humor, participants spoke of trying to find simple articles they could understand:

What I would try to do is I would try to find this… Try, try to find the sort of research that spoke in the most simplest of terms.., once I got halfway through it and I recognized that it was well beyond, above me…(Walter, 49, RN 29 years)

The volume of research available was confusing to them as students and they found it hard to identify which was relevant:

It's so broad trying to get so much, I think I actually did, you know, like I went to areas that wasn't meant to be trying to gather information because of time limit I found it was overwhelming (Fatima, 47, RN 9 years) and evidence for practice was not necessarily connected to research being used for an assignment: I think as an undergraduate, you can't… The research underpins your theory so that you have some kind of extended understandings to what you're doing and why, but once you actually get into a prac experience and you're actually on the floor with your mentors or etcetera, then you kind of don't link the two together (Kathy, 46, RN 11 years).

Early learning also brought with it problems of how to interact with the research literature. How and if to critique the literature was recalled as a significant problem:

I vividly remember thinking, who am I to put up an argument against this? These people have published this, for goodness sake. You know who am I to say that they're wrong? So that was my first thing was it was really difficult (Sophie, 51 RN 30 years)

Developing a critical mindset was not something they found easy to develop:

I just took them all as gospel. You know, what was in these articles was gospel, and I used what I could (Joy, 52, RN 33 years)

Others, however, felt they had personal characteristics that helped them in their early learning years:

I was always a bit of a bookworm, so yeah, I didn't struggle too much with that (Jenny, 52, RN 27 years)

#### Help with learning

A great deal of the focus groups' conversations about their formative years dealt with the help participants had received with their research learning, including help from mentors and role-models. One participant remembered:

When I was doing my nursing degree, one of the best and most memorable tutorials I ever had was in a research topic, which are traditionally the ones everyone hates, find really difficult to do. I had a very inspiring tutor in that topic, and the most memorable tutorial I think I ever had was when we discussed ethics in research (Tess, 42, RN 13 years)

Other participants recalled helpful programs such as peer mentoring, learning success programs, and academic writing courses, as well as library services and librarians that were another source of valuable help. Mentors, lecturers, educators and peers were described as helpful, inspirational, or supportive, and they were described as key to surviving these early learning experiences, according to participants' recollections:

Having good role models, and as I said… Or as I said, mentors, but having mentors, good role models, good people around you that value it helps you to value research 'cause you see what they can make of it (Jack, 55, RN 26 years) and: Study-wise, like I said, I had a fantastic mentor that just encouraged me and pushed me and pushed me, and it was wonderful (Sarah, 59, RN 40 years)

Similarly, the absence of role models was felt to be an additional source of difficulty:

…they tell you to find a mentor or… There just wasn't anyone. You know, it's yeah, a small country town. You don't find anyone, there's, there's nobody that understands it, there's nobody that… that can do that interpretation for you…that…can help you with how to do that (Jenny, 52, RN 27 years)

### Theme 2. Fitting research into the reality of nursing life

This theme and its three subthemes (organizational issues, interpersonal issues, and confidence) emerged from discussions of how reading and using research connected with the rest of their nursing lives. Participants were asked about how any difficulties they had with learning to understand research impacted on how they perceived their chances for success as a nurse, how pressures from their working life impacted on interacting with research literature, and how their degree of comfort with reading and using research influenced their involvement in work activities. The need or desire to read and use research sometimes did not fit well with a nursing career, especially in the early years when it was perceived that consolidating the tasks of nursing was paramount. Supportive structures, senior staff and peers were spoken of admiringly, with a sense that they were 'lucky' to be in a research-friendly environment. Achieving confidence with reading and using research was seen as a function of personal characteristics rather than the actions of educators and workplaces.

#### Organizational issues

A prevalent view across multiple focus group discussions was that organizations were perceived to view nurses' involvement in evidence-based practice (other than simply complying with policy) as an optional extra in the context of getting the job done:

There's really no time for anything else, and from a higher level, research is considered something of a luxury. If there's resource cuts, then education and research are always hit first (Samantha, 55, RN 22 years).

Some participants perceived that preserving the status quo was a higher priority than promoting practice change:

...if people understood how to use the databases, how to research evidence to back up practice or to, or even just to augment their practice great, but it's so hierarchical in nursing and people guard their policies and procedures with their life. I don't think they want change sometimes (Kerry, 53, RN 18 years)

The hierarchical nature of many nursing structures also worked against participants' desires to become involved in EBP activities:

I have never been involved in projects, before because of the hierarchy, I'm at the bottom level (Fatima, 47 RN 9 years)

Many participants worked in organizations with expectations that staff participate in EBP activities, but that did not necessarily mean that resources or support was available to facilitate these activities:

The fact that I was in a, a large metropolitan health service still didn't mean that I could reach out and grab somebody to help me, So but in more recent times, they've put some structures in place to improve that, and it has improved. However, would I call it supportive? I don't know that I'd call it that (Walter, 49, RN 29 years)

There was a consciousness of different organizations being at different levels of engagement with EBP:

…other organizations I've worked for in the past, they're at the forefront, they're engaged with universities and tertiary providers which work alongside the clinical service, and I think that people have a greater understanding about the importance of research and generating research outputs and also using that to inform practice. Whereas, I think that not all organizations are at that stage, which is just how it is really (Ron, 40, RN 16 years)

#### Interpersonal issues

Many participants recognized that EBP was not something they could really achieve alone, and that without the cooperation of their team it was unlikely they could influence practice change. There was also considerable discussion of the overt hostility some had faced when trying to change practice or undertake further studies. The nature of interpersonal interactions was of considerable importance to these nurses, reflecting the strong focus on teamwork in nursing. Being 'different' or acting outside the team's norm put individuals at risk of feeling out of place in their workplace or in their job. Other participants related stories of assistance and support and spoke of their pride in their workplace and team for providing high quality care.

The perception that research and EBP are not really core to nursing was clear from several participants, as one said:

I don't think I actually put the two together as either being the researcher or the clinician nurse, in that I often probably was looking for something because I couldn't find the answer to it. So, I would… Nobody else was looking up anything and so I guess I felt odd, actually (Ella, 34, RN 14 years)

The demarcation between EBP and practice as it happens 'in real life' was made quite clear:

And when you have eight hours to finish everything that you have to get done, the urgent priorities take over the important or even, really don't know if you call it important, I'd call it a side gig(Mei, 35, RN 14 years)

Caring was seen to be at odds with intellectual activity:

…whereas nurses, well, you're supposed to care, like where's where does research fit into that? (Jenny, 52, RN 27 years)

People inside and outside of nursing did not seem to perceive research as something that nurses should be concerned with:

a fairly new RN, who's got a position as a researcher and yeah, she's had a lot of flak from people, including in our family, about, "Why are you doing this? Is that what you did nursing for?" So yeah, it just speaks to the stereotypes about how research is not an essential part of our profession, which of course it is (Jack, 55, RN 26 years)

Supportive teams and colleagues were seen to enable practice improvement through research use:

I don't have much experience outside emergency departments, but I do think emergency and critical care, there is generally a good culture around that sort of thing. When I was quite a junior nurse, for my graduate certificate, I had to do a literature review on pressure area injuries in emergency care. And through that I was able to alter our nursing assessment charts to include a Braden score because of the evidence that I showed the organization about the risks of pressure injuries and things like that. And they were very receptive to that I found (Tess, 42, RN 13 years)

Participants appreciated a supportive culture in the workplace:

So, I've just become interested in research recently, and just talking to people who are in that field in the hospital has been really easy and very helpful and supportive. And yeah, and helping me try to do that in helping you try to learn that as well. So, it's yes. Really, really good. Really supportive (Maya, 30, RN 3 years).

#### Confidence

Participants identified their own personal characteristics as being key to their confidence with research:

I was always very ambitious and thirsty for knowledge. So I read every you know, there are professional magazines that come out like my first place as a registered nurse was the operating theatre. So I read all the operating theatre magazines that came out (Mona, 52, RN 32 years)

Participants related early experiences with reading research that increased their confidence:

I went to search in the library at the [hospital] and got out some articles and read them, and then told my educator that this is what I'm gonna do, and she was of course very impressed. But that was sort of like an automatic. But not all students did that though. You know what I mean? It's probably because I'm just a type A personality and it worked for me… (Diya, 48, RN 20 years)

Confidence with one aspect of using research was perceived as leading to other things:

Yes, I've been taking on, like, you know, the mentoring and the facilitation of the students. And I wasn't really looking into that side of stuff until I started to get a little bit more into the research stuff (Eve, 30, RN 10 years)

Confidence with research literature was something they perceived in other nurses as well:

The nurses who do read articles do stand out, and they're usually of that caliber, and so they're usually in the middle of their Masters or in the middle of pursuing some form of formal education, and even if they weren't, the thing is they're few and far between, that's what I mean by "they stand out," as nurses, the team is receptive to their passion, but they wouldn't be going looking for articles the way this person would (Mei, 35, RN 14 years)

### Theme 3. Working towards using research

This final key theme emerged from the discussions about the participants' experiences with research literature, the feelings they had about using it, and strategies they used for dealing with texts they might find difficult. Four subthemes were identified through repeated readings of the transcripts: approaches to reading and understanding research; using research; mathematics difficulties; and research language. In addition, as a final question to all the focus groups, participants were asked how they would feel if they were asked to read a research paper "right now" and their reactions to that prompt, including their non-verbal observed reactions are discussed.

#### Approaches to reading and understanding research

This was a somewhat unexpected subtheme developed over the course of the focus groups and so was discussed in more detail with the later groups. Participants spoke of how difficult and time-consuming reading research literature was and related their strategies for extracting the meaning, as they understood it, from the papers they read. Very few participants who spoke about their reading strategies stated that they always read the whole article, instead using a range of different approaches.

The methods section of a research paper was a particular source of discomfort, as this participant described in her approach prior to commencing her research degree:

I'd read the abstract and the introduction, skip through all the middle bits, and read the conclusion. None of the actual research methodologies or any of that made any sense whatsoever (Ella, 34, RN 14 years)

Participants developed strategies to allow them to extract some meaning from research articles, even if they had to take the paper's reliability on trust:

...discussion sections were fine as a uni student but trying to interpret what they was talking about in their methods…. And like their results section I kind of skipped past that to the discussion because it was just easier. They even if they were doing something really simple the terminology they used made no sense (Lyn, 24, RN 3 years)

Details of the methods and results were not considered by some participants to be "relevant" to their needs:

I just want to go straight to the facts, I don't care about all that stuff that's probably relevant to a researcher but it's not to me. I tend to go straight to the end to see what the outcomes were and skip everything in the middle, where it's leading to because that stuff just isn't relevant to me on a day to day basis, I just want the information that is relevant (Maryanne, 46, RN 10 years)

Participants also spoke of making pragmatic decisions about reading papers in the context of their limited time:

If I've got the time, I'll read the whole thing. If not, I won't. Definitely being wary of the methodology and the size of the study, and I guess the particular context and any notes on that (Andy, 25, RN 2 years)

They were aware their strategies were not always 'correct' but they were perceived as effective:

Read the abstract content and results. Read the conclusion. That was enough to get through my 3rd year evidence-based practice subject (Eve, 30, RN 10 years)

#### Using research

Many of the participants were undertaking or had completed postgraduate studies and spoke about using research in writing assignments, but they were also using research to underpin practice and to justify their practice choices. They seemed acutely aware of the expectations on them to use research in education and practice, and sometimes these expectations were felt to be burdensome. Despite the difficulties many experienced with understanding research literature, they were still generally willing to try to use it whenever it was needed.

Using evidence to drive practice change in the interests of patient safety was discussed by several participants:

I don't do research. I use research. So, my emphasis is on finding solid stuff to back up things or, you know, what is evidence based on? That's where I'm still quite active in this field of health and safety (Danni, 54, RN 36 years)

There was a sense that proposed change based on strong evidence was less likely to be argued with:

If I put in an improvement form, I'll often staple a couple of research articles to back it up when I hand it in, and highlight what's relevant, and they don't argue anymore (Noni, 54, RN 38 years).

Participants' own personal safety was also seen to be preserved by the use of the right evidence in practice: *Like I work for agency as well. If I don't believe it—if their practices are not based on evidence based practice—I just stick to those places that I know that are evidence based practice because I work in medical oncology/ hematology and I'm very cautious about the fact of how much it will affect me, because I'm still of child-bearing age. So… So, if I work in an area that is not using best practice, I'm not gonna go back there* (Bella, 36, RN 14 years).

For some participants working in education, using and normalizing using research was challenging but necessary:

And so, my challenge has been to try and make it relevant to day to day practice. And it's slow, but it's achievable if you can find projects or links where you can sort of embed a little bit of research in there. And then they say that it's not a mystical kind of weird thing that only a bunch of weirdos do somewhere else (Samantha, 55, RN 22 years)

#### Mathematics difficulties

Difficulties with understanding use of numbers, mathematics, and statistics emerged as a strong theme from these discussions. Participants expressed dismay at the problems they experienced in understanding quantitative results and statistical terminology. Qualitative research, on the other hand, was not considered to be difficult to understand, and the focus of participants' discomfort was centered strongly on numbers and statistics.

Participants found the way that numerical results were written to be confusing:

For me it's the way it's written with all the 0.5 s and all that sort of thing, it doesn't make sense. If it was simple percentages, then that makes sense (Joan, 60, RN 30 years)

There was a sense that statistical terms were a language they did not speak:

...just enough on stats. I think there's something a bit harsher about them being a bit more numbers, but thing I hate about them is almost that foreign language involved, you know, squared chi Wilcoxon and whatever the hell of the names of the and so they frighten me a bit (Sally, 50, RN 8 years)

One participant queried whether discomfort with numbers was related to gender:

It's feeling comfortable with using numbers and whether that's a male or female thing, talk about it as gender, but just feeling really more comfortable, with say, phenomenological studies and things like that just seem to make more sense, and whether that's why I'm a nurse or it's..[trailed off] (Gen, 65, RN 48 years)

However, male participants expressed discomfort also:

The second I saw like, you know, the analysis and all that kind of stuff, I'm like I'm not gonna read over this, you know, You see that I'm not a very numbers person (Bob, 48, RN 1 year)

There was a sense that numbers and feelings were diametrically opposed:

I much prefer to read a qualitative paper… Yeah, rather than… I'd rather read about people's feelings, than the numbers (Joan, 60, RN 30 years)

Numbers were seen as excluding the human element that nurses value:

I also think it's about whether you like the human element and people mattered more to me than numbers. I think it's maybe that and probably I think, you know, when I went to midwifery and child health, that's all about more about humans (Lisa, 54, RN 33 years)

#### Research language

The specific language used in research was a problem for many participants. They seemed alienated by the language; despite the often-complex terms used by their various clinical specialties the terms used in research seemed untethered from logical meaning. That lack of connection to an action or object that could be clearly conceptualized meant that participants often felt that research was not written with them as readers in mind. When they could see a clear connection to their work or studies, research language became more relatable and easier to understand.

Research language was viewed as alien or foreign:

I think there's an aspect of unfamiliarity with the language too, because it's like reading anything in a foreign language, it's really hard work. And to a lot of nurses, research is a foreign language. They're not being exposed to it (Jack, 55, RN 26 years)

There was a strong sense that research was genuinely regarded as language not everyone could speak:

I haven't done research, so I can talk about research I've read with people at work, but it's like talking another language (Noni, 54, RN 38 years)

Trying to understand the language was full of pitfalls:

So, I started in that levels of hierarchy and evidence. I started then really starting to get picky about what I was really and looking at the language then got confused with intervals and confidence of a lot of talk about 0.95 (Eve, 30, RN 10 years)

Particularly in their early years, it was difficult to engage with research literature due to the language:

I lost interest straight away… I'm better now than I was then, obviously, but in those days, yeah, I was absolutely intimidated by the, the way it was written (Walter, 49, RN 29 years)

The language used in the paper was tied to how much effort participants would put into trying to understand it:

…it was so full of so much jargonized rubbish, that you almost needed to research that research paper, whereas then you find another person who's writing it in a tone or a language that you can understand and you immediately resonate (Kathy, 46, RN 11 years)

Difficulties understanding the language also influenced their reading strategies:

It's a discussion section that I go to. First, the abstract, but then after that the discussion, and only if it's got anything useful, then I will go further if I have to, but that's because the plain English is in the discussion section, that's where they don't dribble on about X equals Y, and we found that, blah, blah, and the average of this was that and… Yeah, 'cause I understand they have to spell out their tables and Excel tables and findings and everything. But the discussion is where the English is, that's where normal human speak is (Mei, 35, RN 14 years)

Despite these issues, most participants, when asked how they would feel if asked to read a research article "right now", responded at least somewhat positively. Some conveyed considerable wariness or concern in the tone of their responses:

I would want to know what the topic was and I would want to know. I would want to know why you wanted me to read it (Nina, 57, RN 9 years)

Some responded with defensiveness:

Again, why? I've got plenty to read. I don't need what you want to give to me to read. Is there any benefits in this particular paper? What is it trying to achieve? So is it a valid study or is it just some ivory tower, need to know something for the sake of it? (Anna, 59, RN 19 years)

Even with a hypothetical request, participants were cautious about committing their time:

I'd be more likely to actually be able to get through it if it was a shorter one rather than a 20 page (Karen, 35, RN 13 years)

Most, however, responded with confidence they would give it a try:

I'm gonna say yes. Tell me what it's about, and I'll say yes, let's read it and see what we can do (Diya, 48, RN 20 years)

## Discussion

Participants in this study responded with a rich variety of stories about their experiences and how they felt about reading and using research literature. Some participants were, as described in the literature, 'research reluctant' [28] but many held positive views. Having positive attitudes towards research and EBP did not mean participants experienced no challenges with reading and using research, however. Positive attitudes to EBP, combined with involvement in research education and activities, regular journal reading, and higher levels of education have been found to be associated with higher levels of EBP engagement [2, 3], but engaging nurses in those educational activities and promoting higher education can be a difficult task.

We deliberately avoided framing the focus group discussions in terms of barriers and facilitators, largely because for more than 30 years a segment of the nursing literature has framed the question of nurses' engagement with EBP and research in terms of this binary [29–31] with little progression in resolving this issue. Barriers and facilitators, while conceptually helpful in considering issues of implementation, are less so in the presence of an unclear and complex situation such as this. It was also important to gain a deeper understanding of the issues rather than simply statements of barriers or facilitators.

Research methods education at the tertiary level is often designed to train students to conduct research, whereas in most clinical fields such as nursing, the majority of students will be research users [32]. A systematic review of non-discipline-specific research methods education studies presents some findings similar to the perceptions and experience related by participants in this study [32]. Earley’s review synthesizes a number of student characteristics observed in the 51 included studies, such as “They are typically anxious or nervous about the course,” “They fail to see the relevance,” and “They come to the course with poor attitudes about research,” [32](p. 245).

This study adds several nuances to the current conversation about nurses' EBP and research engagement. In exploring the research reading strategies used by the participants this data connects with other work conducted on research reading strategies [33]. Similar to the findings by Hubbard and Dunbar [33], their sample of undergraduates and early career researchers in biological sciences placed less value on understanding the methods and results sections of a paper, as did many of the nurses in our study. Some participants in this study believed the methods and results sections held little useful information for them. It has been suggested that addressing research language difficulties can help increase engagement and improve reading strategies [33].

Research language has been identified as an issue for learners across the professions, including nursing. Nurses in a Swedish quantitative study were asked several questions about their experiences reading research literature, with the vast majority indicating they only "sometimes" understood the research articles they read, and that if research articles used "simple/normal language" they would read them more often and apply the findings in practice [4]. Participants in our study also commented on their difficulties with the language in research papers and expressed a wish for simpler language to be used. As research writing conventions are unlikely to change, it may be that a different pedagogical approach would be beneficial for bringing learners into an understanding of research literature. Learning the language of research has been compared to second language acquisition and the use of similar teaching and learning approaches has been suggested [34]. A language-based approach, genre analysis, has been piloted with registered nurses for research methods education with some success, however more work is needed [35].

Related to participants' difficulties with the methods and results sections of research papers, may be connected to a well-known phenomenon known as mathematics anxiety – a fear of or aversion to mathematics, which often leads to avoidance of mathematics-related activities [36]. Participants in our study made many mentions of "the numbers"; they felt numbers were hard to understand, incompatible with caring, and confusing. Given the importance of mathematics to nursing, any changes to research methods pedagogy will need to include strategies to improve attitudes to and abilities with understanding and interpreting numerical reporting in research literature [37].

In our exploration, we focused on nurses' experiences and the feelings they attached to those experiences, rather than research attitudes or knowledge, although both these are important, they are not the whole story. Whatever the sources of the challenges in addressing nurses' engagement with research and EBP, it seems clear that a multifaceted approach is needed. Effective pedagogies along with strategies to address work culture and organizational challenges are all needed to provide the environment for evidence-based healthcare to flourish.

### Implications for practice

Some of the factors influencing nurses' perceptions of research, such as mathematics anxiety, may not be modifiable by nursing educators at a tertiary or workplace level, however confidence and self-efficacy in terms of reading and understanding research can be increased by creating success experiences using effective pedagogies [38]. Creating scaffolded research methods education that gradually introduces nurses into an understanding of research literature focusing on both understanding the language and understanding the statistics and numerical reporting may be the most appropriate approach to creating familiarity, and increasing self-efficacy, therefore leading to better experiences and greater engagement. Increasing the research friendliness of workplaces and availability of mentoring options would encourage all nurses to engage with research.

### Implications for research

There is likely to be considerable value in investigating new pedagogical strategies for teaching research, both to undergraduates and registered nurses. Future research could further investigate in detail the theorized link between research aversion and mathematics anxiety.

### Limitations

Registered nurses who self-selected to participate in this study may have been systematically different in important ways from nurses who declined to volunteer, particularly in their level of education and interest in research. Many of the participants were in senior roles in their organizations, and some were studying for research degrees. Participants in this study were slightly older than the average Australian RN (44.3 years vs 47.5 for this study) and slightly more likely to be male – 89.1% of Australian RNs are female, while 87.5% of these participants were [39]. These small differences may affect the transferability of these findings to the wider population, however the findings do align with other work, such as that by Hendricks and Cope [4], the findings of which are discussed above.

## Conclusion

Nurses have a wide range of experiences interacting with research literature, but many report struggling with the language, the numbers, or the statistics. Many nurses value research and EBP and capably use it in practice, however the current reading strategies used by nurses in this study do pose a risk to EBP if research is used without being properly appraised. Nursing workplace cultures are a significant influence on how nurses perceive research reading and use, and workplaces with hostile or apathetic culture toward research risk poor practice and alienating staff interested in improving practice.

## Data Availability

The data used in this study are available from the corresponding author on reasonable request.
